# De L’Influence De La Navigation Et Des Pays Chauds, Sur La Marche De La Phthisie Pulmonaire

**Published:** 1857-11

**Authors:** 


NORTH AMERICAN

MEDICO-CHIRURGICAL REVIEW.

NOVEMBER, 1857.

### Analytical and Critical Reviews.

ART. I.—*De l'Influence de la Navigation et des Pays Chauds, sur la Marche de la Phthisie Pulmonaire.* Par M. JULES ROCHARD. Second Chirurgeon marin au Port de Brest. (Mémoires de l'Académie Imperiale de Médécine, T. XX.)

On the Influence of Sea-Voyages and Warm Climates, on the March of Phthisis Pulmonalis, &c. &c.

*On the Effects of Climate on Tuberculous Disease.* By EDWARD LEE, M.R.C.S. London. Fiske Fund Prize Essay. In the American Journal of the Medical Sciences, April, 1857.

*Le Climat de l'Italie sous le Rapport Hygienique et Medical.* Par le DOCTEUR ED. CARRIERE. A Paris, 1849, pp. 582. 8vo.

The Climate of Italy in its Hygienic and Medical Relations. By ED. CARRIERE, M.D.

*On the Curative Influence of the Climate of Pau, and of the Mineral Waters of the Pyrenees in Diseases.* By M. A. TAYLOR, M.D. Pau, 1843, pp. 437. 8vo.*

THERE are two propositions which may be laid down and affirmed at the present time, regarding Pulmonary Consumption. They are, 1, that little is known respecting the sanative influence of climate on this disease;

* We avoid the seeming affectation of giving the title of the French translation of this work, which we have in our possession, and of which we make use; not knowing, when we procured it, that the original was in English. This last we have seen but have never read.

and, 2, that it is curable, for it has been cured, but by what means, or under what circumstances precisely, cannot be said. By far the greater number of instances of this favorable nature have been brought about spontaneously, or under circumstances not represented by any definite, or at the time recognized hygienic or therapeutic agencies, such as could be repeated with any prospect of similar results. In reference to the first proposition, if an acknowledgment of ignorance be the first step to knowledge, we may entertain some hopes of a better acquaintance with the real operation of climate in phthisis. Hitherto, or until quite recently, the process of reasoning, and the practical deductions from it, were of a purely negative kind. As a residence in a country, the climate of which was distinguished by certain obvious characteristics, was found to be prejudicial, by its causing and aggravating phthisis, it was inferred that removal to another country, the prominent traits of the climate of which were different from the first, must necessarily be beneficial; and, accordingly, the patient was sent on his journey, buoyed up with hopes of cure, but which, in the majority of cases, were found to be utterly fallacious. There were once persons in England, silly enough to believe that, when they crossed the Straits of Dover, and had fairly got into France, they had made a beneficial exchange of climate; and that their weak lungs would be improved by a change from London to Paris. They were ignorant of the terrible frequency of both acute and chronic diseases of the lungs in the French capital; and of a state of atmosphere, there, during the winter months, far more trying than that which they had left behind; while they encountered an absence of domestic comforts and house arrangements which they had enjoyed at home. We are afraid that this class is not yet entirely extinct, and that it is, to a certain extent, kept up by the credulous from our own country, who, in the midst of the pleasures and gaieties of Paris, try to persuade themselves that they are gainers also on the score of health. Perilous mistake, to drain the cup of Circe, as if it were a draught from the fountain of Hygeia!

For the last century and a half, invalids with weak chests, and sufferers from chronic pulmonary disease, and especially phthisis pulmonalis, have been in the habit of visiting the south of France, and spending the winter there. How little they were guided in this measure by positive knowledge and experience, was evident from the selection of Montpellier, the name of which, for a long time, was used as synonymous with a healthy spot, enjoying a pure and sanative air. How such a reputation came to be produced passes understanding. As this town was, for a long time, the seat of a celebrated school of medicine, some of the teachers in the latter may have persuaded the credulous youth from other countries, and particularly that portion of them from England, Holland, and North Germany, that while they were imbibing medical lore, they were also inhaling a genial and exhilarating air, and receiving in their own persons the double gifts of Minerva and Apollo, the latter of whom it is known, is sometimes regarded as the tutelary deity of medicine. Whatever may have been the origin of the belief, one thing is certain, that it took firm hold of the minds of all Europe, notwithstanding the occasional dissent of such popular writers as Sterne and Smollett among the English. Smollett made grievous complaints of the deceptive views held out respecting the climate, not of one spot only, but of Provence entire. Sterne, who had been subject to hæmoptysis and pleurisy, writing from Montpellier, February 1st, 1764, says: "I have been dangerously ill, and cannot think that the sharp air of Montpellier has been of service to me; and so my physicians told me when they had me under their hands for above a month. 'If you stay any longer, sir, it will be fatal to you.' 'And why, good people, were you not kind enough to tell me this sooner?'" A very natural question, which might be enlarged for the benefit of physicians everywhere, by their being asked also, "Why not, learned gentlemen, make yourselves acquainted with the climate of the place in which you live and practise your profession, so as to be able to tell invalids what they may expect by taking up their residence or prolonging their stay among you?" The advice dates as far back as Hippocrates, who, old fogy as he may be regarded by young medicine, has uttered many good things besides this. Thirty years ago, or more, when lecturing on the climate of the Mediterranean in phthisis, we used the following language: "Of Montpellier, we no longer speak as a residence for persons in consumption, any more than of Aix or Avignon. The physicians of these places send away patients laboring under the disease. To inculcate, however, the extreme importance of attending to the circumstances of aspect, as regards the exposure of a place to particular winds, we may mention that Montpellier is beautifully situated upon a hill: the town surrounds and covers it, and of course it has a northern as well as a southern aspect. The physicians and inhabitants are well aware of the difference presented in these exposures. It is on the southern side alone that the invalid is permitted to promenade; and where health is in danger, a house is selected, sheltered from the keen northern blasts." We were, perhaps, premature in our conclusions, that Montpellier was no longer spoken of as a residence for the consumptive. We fear that the delusion on this point is not entirely dispelled, even at the present day, and that there are not wanting some persons, who, in their early life, had read of Montpellier, and still put faith in such language as that used in a work prepared with so much care as the *Encyclopedia Americana*, 1832, viz.: as a town "particularly celebrated for the salubrity of the air," and "much visited by invalids from foreign countries."

A better knowledge of Montpellier climate might be procured, even by retrospective reading, in some other directions than the common and popular one. We can hardly expect this, however, from general readers, or even scholars, when the professional ones are so little inclined to such an exercise of their faculties. As far back as the year 1805, M. Baumes, in a work on pulmonary phthisis, expressed himself in the following terms: "The north is the most prevalent wind in winter and spring; its force is often very uncomfortable, for it is very cold, after its passage over the snow of the mountains adjoining. One must have a well-constituted chest to resist its effects." Five years later, or in 1810, in a topographical notice of Montpellier, by Muret, it is stated, that out of 2750 patients admitted into the Hôtel Dieu, the chief hospital of the town, in 1763, there were 154 deaths, of which 55, or more than one-third, were from phthisis. Well might poor Sterne express his disbelief in the sharp air of Montpellier having been of service to him. We will not say *ab uno disce omnes,* but we do say that the undeserved reputation which one town in the south of France acquired, as a proper residence for consumptive invalids, should make us slow to put faith in the reputed favorable hygienic conditions of other places, either in this region or south of the Alps, in Italy itself: at least it should induce us to scrutinize rigidly the climatic elements which can entitle them to our confidence.

Climatology, bearing any semblance to a scientific inquiry, and advancing beyond the ancient division of the earth into zones, and some empirical observations made at different times in certain localities, can only be said to date from the labors of Alexander Humboldt, in which he was aided by Von Buch. Among later laborers in this field, the names of Dove, Boudin, and Blodgett, deserve more conspicuous notice. The isothermal lines which he indicated, and in part worked out, were substituted, in the study of climatology, for lines of latitude, with which they not only are not parallel, but do not, in many regions, correspond with each other. One great step was thus made in showing that places in Europe and America, for example, differing by many degrees in latitude, have the same medium heat throughout the year, or are on the same *isothermal* line. Thus, for example, Philadelphia and London, separated from each other by eleven degrees of latitude, are nearly on the same isothermal line, or that of 50° F. But even this fact being established, must not lead to an inference that they possess an identical climate, or that their vegetable productions are the same. A very hot summer and a very cold winter in one, may give the same mean annual temperature as in the other, which has a summer less hot, and a milder winter. And again, places are found to have a like summer temperature, or to be on the same *isotheral* line, without their being on the same isothermal one; and places may have their winter of the same temperature, or be on the same *isocheimal* line, and yet be on different isothermal as well as isotheral lines. It is very evident, from these new and more numerous data for forming a better judgment of the climate of a place, and for instituting a comparison between it and any other, either with a view to discover a resemblance or to detect a difference, that a mere knowledge of the mean annual temperature is only an element in the calculation. It would not serve us in better stead, as indicating or even suggesting fitness for the abode of an invalid, than if we had saved ourselves all the trouble of daily thermometrical observation and record, and reached the same result by simply ascertaining the temperature of the springs, or of the wells of the place under examination. But if we ask for the difference between the temperature of what may be called the winter half of the year, or that from the 1st of October to the 1st of April; and of the summer half, or that from the 1st of April to the 1st of October, then will the annual register be of some value, and help us in our inquiries for a winter residence suitable to an invalid with weak or positively diseased pulmonary organs. We say help, for temperature is only one of the elements that make up climate. The hygrometer has to be watched, and its changes noted, so that we may form an idea of the relative humidity or dryness of a place, about the salubrity of which we would inquire in our hygienic tour of inspection. Yet another and a very important element, perhaps we ought to say condition, since it is compounded in part of temperature and in part of the hygrometric state of the air, comes up for observation and study. We mean the prevalent winds, and the force with which they blow. The brow of a hill, a wall even, or a piece of woods, by sheltering an invalid from the keen northerly wind, will place him in quite a different climatic condition from that in which another finds himself on the northern or windward side of these screens, notwithstanding that the medium temperature in which these two persons live may be the same. It is chiefly by such a screen as this, but consisting of a continuous range of lofty houses, that Pisa owes its reputation as a winter residence for consumptive invalids. So, on the other hand, what important modifications in climatic influences, operating on an invalid, are produced by a prevalent southerly wind, particularly on the coasts of the Mediterranean. The various states of the electricity of the atmosphere constitute another, although less readily appreciable element, physiologically considered, in making our acquaintance with climate.

The problem to be solved before we can select a fit residence for an invalid, or recommend him with propriety to leave his own for a foreign land, or even to change his abode in his native country; as for instance, to go from a Northern to a Southern State, or from an Eastern to a Western one, is exceedingly complex; the more so, indeed, for the reason, that, although we may be said to know the elements, we cannot yet appreciate in a satisfactory manner their combination or fusion into what we call climate. The effects of climate are, also, as complex as its composition; and even while we talk of the sanative influence of the air of a particular place on the enfeebled or diseased lungs of an invalid, we cannot forget that this sanative influence, to be effectual, must also be exerted on the digestive and nervous systems, and on the cutaneous as well as on both the respiratory and digestive mucous surfaces. Can we always seize, in our minds, on the atmospheric condition, which allays a marked irritability of the heart, and diminishes the frequency of the pulse, or carry away with us such a definite knowledge of this condition as to serve us for a standard by which we can ascertain the existence of a similar atmospheric condition and character in another spot? While replying in the negative, we are obliged to go a step further, and admit that our practice, so far, in prescribing subjection to new climatic influences is a good deal empirical. Then comes another—a final question: whether, if we could procure access to an air of singular purity, consisting of all the requisite qualities in due proportion, it would be adapted to maintain our organism in its greatest vigor, or to restore it to health when it had been suffering from chronic disease, phthisis or scrofula, for example? Might we not be as much disappointed as we were when, upon procuring pure water by distillation, and thus deprived of all foreign ingredients whatever, the discovery was made of its unfitness to nourish animal beings. Poetry may describe a land equally protected from rude Boreas' blast, or Auster's sultry breath, and over which the zephyrs sport, while the earth is redolent of sweets and balmy odors, and clothed with vegetation of every hue; but it is not in such a land that the body would attain its greatest vigor, or the mind display its highest and most diversified energies. The hardy and bold men of the North, who overran and subjugated the Roman Empire, were nursed in storms, and exposed to constantly recurring vicissitudes of weather. When lulled to repose, it was by the moan of the chilling blast through their vast and gloomy forests.

Climatology includes not only a study of the climates of regions and countries, and a comparison of the climate of one with that of another, but also of limited districts and spots, between two of which, even at a short distance from each other, there are not unfrequently strong climatic differences, depending on their relative proximity to mountains, maritime exposure, the nature of the soil, &c. Thus, for example, the southern and southwestern coasts of England enjoy a far milder winter climate than the midland and northern counties of that country; and in this maritime belt there are spots still more favored than the rest, and which may be assimilated to some of the chosen retreats for invalids in the south of France and Italy. Of these favored local climates, that of Penzance, on the south coast of Cornwall, claims the preference; but for reasons which illustrate the necessity, in the study of climatology, of attending to the medium temperature of the different seasons rather than that of the whole year, and of the night as well as of the day. "The mean annual temperature of Penzance is 52·16°, being only 1·77° above that of London. But the temperature is very differently distributed over the year at the two places. Although Penzance is only a degree and a half warmer than London for the whole year, it is 5½° warmer in winter, 2° colder in summer, scarcely 1° warmer in the spring, and only 2½° warmer in the autumn."* The greater part of the difference of temperature in winter between these two places is in the night, during which Penzance, on an average, is six degrees and a half warmer than London, and during the day it is only a little more than three degrees warmer. The equal distribution of heat throughout the year shows, by comparison with the south of Europe, the climate of Penzance to still greater advantage; and in this respect Madeira and the Azores are, of the climates examined by Sir James Clark, the only ones superior to Penzance in this particular. The daily range at the latter place is little more than half that of the south of Europe.

Carrying out the study of the climate of Penzance in other seasons, we find that it loses its superiority in the spring. "In April and May it appears decidedly inferior to the more sheltered spots on the south coast of Devon, and to Undercliff (Isle of Wight), and very much so to the southwest of France. For instance, at Pau, the mean temperature during the winter is nearly 3° below that of Penzance, while during the spring it is 5° above it." Among the drawbacks to Penzance as a winter residence, are the humidity of the atmosphere, there falling in it about twice as much rain as at London, and its liability, in common with the whole southwestern extremity of the island, to violent and frequent gales of wind. One of the effects of these latter is an elevation of temperature, most evident during the night; so that the difference in this respect between the day and night is very slight. During these *moist siroccos*, which are so prevalent in winter, the inhabitants may be said to breathe the breezes of a climate milder than their own. When the south and southwest winds are very gentle, and the days are clear, the warmth and softness of the air are delightful. The relatively greater coldness of Penzance during the spring months is owing to its exposure to north and east winds. Sir James Clark judiciously remarks on this occasion: "The circumstance of exposure to or shelter from cold winds, constitutes the principal cause of the difference of different places, in the same line of climate, in point of warmth, as

* Clark on the Sanative Influence of Climate.

experienced by man; for the influence of temperature on the living body is indicated much more accurately by our sensations than by the thermometer. Unless, therefore, the indications of the thermometer are corrected by observing the winds, we shall form very erroneous ideas of the climate of many places."*

Pau, just now mentioned, furnishes another example of a local climate distinguished by striking peculiarities, that do not belong to that of the country of which it forms a part, nor even to the section to which it more evidently belongs. A mere knowledge of the temperature of this place would help us but little towards an estimate of its fitness for the residence of invalids. The modifications according to the season, and the daily range of temperature, are important considerations; but even these would not furnish the necessary data, which must be completed by a notice of its hygrometric condition and the prevalence of particular winds. In this last respect Pau is in strong contrast with Penzance, in its freedom from high winds, and in the general calmness of the air, which is a striking feature in its climate. "The mean annual temperature of Pau," as we learn from Sir James Clark, "is 4½° higher than that of London, and about 3° higher than Penzance; it is about 5° lower than that of Marseilles, Nice, and Rome, and 10° lower than that of Madeira. In *winter* it is 2° warmer than London, 3° colder than Penzance, only 2½° colder than Marseilles and Rome, and 7° colder than Madeira. The range of temperature between the warmest and coldest months at Pau is 32°; this, at London, and likewise at Rome, is 26°; at Penzance it is only 18°, and at Madeira 14°. The daily range of temperature at Pau is 7½°; at Penzance it is 6½°; at Nice 8½°; at Rome 11°."

Pau, formerly the capital of the province of Bearn, and the birthplace of Henry IV of France, is now the chief town in the department of the Lower Pyrenees. It is situated in about 43° 20′ north latitude, and nearly in the same line with Montpellier, Marseilles, and Nice, all of which are between the 43d and 44th parallels, and consequently a degree north of Boston; but what a difference between the climate of this last and that of the French towns! The isothermal line of 60° F. passes through Pau, and, on our coast, through Norfolk, and, in the Pacific region, Sacramento; while the isothermal line of 40° would pass through Boston on this continent, and London and Amsterdam on the other. Pau is about 150 miles from Bordeaux, and 50 from Bayonne, on a terrace formed by a ridge of gravelly hills, overlooking an extensive valley to the north. The Pyrenees rise gradually behind it, their higher range being nearly forty miles distant, and exhibiting clearly some of their lofty peaks, such as the *Pic du Midi de Bigorre*, the *Pic de Ger*, and *Pic du Midi d'Ossan*, the glaciers and

* Clark, *op. cit.*

the mountains which inclose the thermal springs of *Eaux Bonnes* and *Eaux Chaudes.*

It may seem paradoxical to those unaccustomed to investigate the subject, when it is said that the air of Pau is dry, although the quantity of rain which falls there is greater than in London, the proportion being as 40 to 42 inches in the former to 30 inches in the latter. The number of rainy days is less, however, in the French town than the English capital, being as 109 to 178. The rain falls in a greater body at one time, and usually a little before or a little after sunset at Pau, and seldom more than two days at a time. Owing to the absorbent nature of the soil, and the declivity of surface by which the rain not thus disposed of flows off, there are few days, as we learn from Dr. Taylor, in which a person in good health cannot pass two or three hours in the open air, and in which even invalids, provided they are warmly clad, may not take some exercise, towards the middle of the day. The westerly wind, which is the bearer of rain, does not bring cold with it; and it is a matter of general observation, that what is called bad, that is, wet weather, calms the cough resulting from tubercles, or acute irritation of the bronchiæ, diminishes the frequency of the pulse, and causes a general feeling of better health. The most genial wind is from the southwest, which is attended with the good effects of that from the west, but it is without rain. The north wind blows with but little force, and is not frequent; and the oppressive southerly winds are of rare occurrence, seldom continuing beyond twenty-four hours. While thus exempt, in a great measure, from these last-mentioned winds, on the one hand, it is equally so from the cold northeast winds on the other,—both of which prevail over this part of France generally. The north winds in the winter at Pau are spoken of as warm rather than cold ones; for it must be borne in mind that this place is to the north of the Pyrenees. In fine, so protected is Pau from winds, that it is not easy for the inhabitants to tell from what direction they blow; and hence, owing to the absence of any great perturbation of the atmosphere, its numerous variations are inoffensive to the invalid.

The mean temperature of the day, from observations made at 9 A.M., 1 P.M., and 5 P.M., or during the period of the twenty-four hours when invalids may be supposed to take exercise in the open air, is for the autumn 62·2° F., for the winter 46·2° F., and for the spring 57·7° F. It was observed, says Dr. Taylor, that during the winters of 1838–9, and of 1840–1, those laboring under affections of the chest could without danger expose themselves to the open air in the months of November, December, and January.

The characteristic quality of the climate of Pau is the comparative mildness of the spring, and its exemption from cold winds. It seems to exert a decidedly sedative effect on the natives, in its causing a predominance of the phlegmatic temperament, and a relatively slow circulation and also slowness of thought and of movement. On strangers a similar operation is manifested, in a diminution of nervous irritability and reduction of the activity of the circulation, and also in a desire for rest and enjoyment at the present, without any anxiety for the future. These feelings result from a removal of organic irritation, one sign of which is a more equable, soft, and slow pulse. Hence we find claimed for the climate of Pau a tranquillizing effect on those of a sanguineo-nervous temperament with its often resulting convulsive disorders, while it exerts a directly sanative influence on chronic inflammation of an organ, a state implying both previous sanguineous and nervous irritation. It prevents, also, Dr. Taylor alleges, the formation of tuberculous matters, and arrests, in a measure, their growth to maturity. Dr. Clark, on the authority of Dr. Playfair, who resided several years at Pau, says, that the mildness of the spring, and its little liability to winds, render this place favorable to chronic affections of the larynx, trachea, and bronchiæ. Dr. Playfair found it beneficial in gastric dyspepsia, and he has seen it useful in a few cases of asthma. The climate agrees well with delicate children, who often acquire good health and greatly improved strength and vivacity, especially if they are removed to the mountains during the summer. Upon the whole, Pau appears to Sir James Clark to be the most desirable winter residence in the southwest of France for invalids laboring under chronic affections of the mucous membranes. Dr. Taylor, however, with all his admiration and eulogy of the place, points out morbid conditions in which the climate would be injurious. These are, all diseases in which there is nervous congestion and diminished energy of the nervous system,—in atonic dyspepsia and the train of symptoms which accompany it; constitutions enfeebled by long residence in hot climates, and in which the functions of the liver are weakened to the last degree; the catarrhs and chronic bronchitis of old persons, marked by diminished tone of the organs and excessive expectoration; chronic rheumatism, with debility of the digestive organs, and complicated with atonic gout; predisposition to apoplexy, owing to passive congestion of the chief organs in a leuco-phlegmatic temperament; and chlorosis with an atonic state of the functions and congestion of the uterus.

We have been led into some details in speaking of the climate of Pau, from its being less known than other favorite spots in the south of France and in Italy, over some of which it would seem to have decided advantages for the invalid. The scenery around is varied and romantic in its features. There is also another recommendation of Pau as a winter residence, viz., its being in the neighborhood of the numerous active and celebrated mineral and thermal springs of the Pyrenees, which the invalid can readily visit during the months of June, July, and August, and thus continue, by their use, under judicious selections and advice, the sanative course begun at Pau during the nine preceding months, or from the first of September to the first of June.

Desirous that our countrymen—traveller sand invalids—should look in this direction for health, we ought to add that the time spent in seeking it while inhaling the sanative air of Pau, need not hang heavy on their hands when they are there. The town contains about 14,000 inhabitants, and is surrounded by a country which invites to excursions in different quarters. The promenades in the environs are very fine—some of them along the rapid river Gave, which passes by the town, others on a smaller stream; and even in the town itself, there is a platform or terrace, well shaded, and commanding a beautiful view. But, of all, the "Park" is the principal promenade. It consists of a terrace, a quarter of a mile in length, overlooking and parallel to the river, and thickly planted with trees. Ladies can always enjoy their walk here, provided there have been three hours sunshine after even thirty-six hours of rain. It is intersected by walks; some straight, some winding in all directions, as if to gratify the various tastes of strangers of every country. The town itself boasts of its *château* or castle, the construction of which dates from the thirteenth century. It is held in great veneration by the Bearnois, on account of Henry IV having been born there. A three minutes' walk brings the visitor to a small two-story house, in which Bernadotte, one of Napoleon's marshals, and afterwards king of Sweden, was born. Pau has also a public library, containing 15,000 volumes on all subjects. It is open the greater part of the day, and every facility is given to readers to consult the books on its shelves. There is also a college, erst royal, now imperial. Boys may be placed here as boarders, if it is desired, and proper attention is promised to be given to their health. Nor are the means for private instruction wanting. In a commendable spirit of toleration, although the institution is of course Catholic, no interference is attempted with the religious tenets of the pupils. A Protestant minister visits the college regularly, and examines the youth of this persuasion, who are also taken every Sunday to church. A commodious edifice has been built by the English residents and visitors for those belonging to the English Episcopal Church, in which service is performed every Sunday, twice in English and twice in French. Among the places of amusement may be mentioned a large hippodrome, which is a good deal frequented. During the winter *soirées* are frequent, and at them one meets with very agreeable company.

Exercise is taken on foot, horseback, and carriage; advantages not enjoyed fully in large towns, the wearisome suburbs of which have to be traversed before one can reach the open country. Five different routes diverge from Pau, exhibiting great variety of prospect of mountain and plain. The equestrian can take a wider range still, and skirt the hillsides and penetrate the forest glades.*

Our first reference to Pau in connection with climatology having been followed, without any prior intention, in this stage of review, by a somewhat methodical description of that town, as a winter residence for invalids, we may as well complete our notice of Dr. Taylor's work by a very curt reference to the contents of the chapters which follow. Of these are a comparison of the climates of Montpellier, Hyères, Nice, Rome, Florence, Pisa, Naples, the Baths of Lucca and Sorrento, with Pau, into which we shall not enter, but reserve to ourselves the privilege, if room is left us, of making some remarks on the sanative reputation of these places as we advance. Reflections on the climate of the earth in olden time, the upheaval and physical appearances of the Pyrenees, and descriptions of strata, the flux of the springs and the intermediate valleys and mountains, and the natural history of the Pyrenees, make up the remainder of the first part of the work. The concluding part and half, on the Pyrenean Thermal and Mineral Springs, will not engage our attention at this time. The theme is fruitful; but its treatment would lead us away from our present subject, the various bearings and details of which require a careful scrutiny.

The complex problems of the true climatic character of the different places recommended as desirable abodes or stopping-places for those suffering under chronic diseases, and especially phthisis, having been stated, we are now better prepared to hear certain fallacies exposed and illusions dispelled respecting their alleged efficacy in this way. Much has been done in different quarters in aiding such an exposure. One of the most com-

* As there are few travellers who have not some curiosity about the rates of living abroad, we subjoin a few particulars on this head, which we find in Dr. Taylor's volume. Some of the *palais* of the old families, who have become extinct, and many other mansions, both in the town and its environs, contain numerous *apartments*, or suites of rooms, ready furnished, which can be procured at prices ranging from five hundred to seven hundred dollars, for eight or nine months. Others again are rented at from two hundred and fifty to five hundred dollars. Some of these suites are spacious enough for the accommodation of twelve to fifteen persons, and all those of the first category have room for from six to eight persons. Plate and house linen are procurable by hire in the town. At the hotels, either a suite of rooms or a single room can be had; or travellers may lodge in a private house and eat at the hotel. They who prefer to keep house can obtain a good cook at three or four dollars a month,—less than we pay in our own country for a bad one,—and a servant man for six or seven dollars a month. Carriages are let at the rate of forty cents an hour, and saddle-horses for half a dollar a day.

prehensive and direct arguments, and which is sustained by the introduction of a large body of facts and observations on the twofold theme of the effects of sea-voyages and warm climates in pulmonary phthisis, is that furnished in the Memoir of M. Rochard, which is the first of the works whose titles head the present article.

The author first adverts to the very ancient belief in the efficacy of sea-voyages in pulmonary consumption,—a belief echoed in the last century by Boerhaave, Cullen, Fothergill, Gilchrist, and others; and he then suggests a plausible explanation of its continuance for so long a period, viz., in the imperfect diagnosis of diseases of the chest, so that chronic catarrh and bronchitis were confounded with phthisis, and cures of the former were regarded as cures of the latter. Laennec had great confidence in the sanative powers of sea air. Knowing the tuberculous state of his own lungs, he abandoned Paris and practice, and went to his native Brittany, where he died on the borders of the ocean. M. Rochard collected 165 observations of cases of phthisis showing itself at sea among ships' crews. Of these, 103 died on board, and 62 were sent home to France, or left in the hospitals of the colonies. As to the alleged infrequency of phthisis on the coasts of Brittany, which, it must be remembered, are exposed to the western winds from the Atlantic, and so far are in the enjoyment of sea air of the best kind, the author gives us a part of the vital statistics of the seaport of Brest. In the year 1853 there were 1519 deaths in that city, 245 of which were due to phthisis. The population is 63,000 inhabitants, including the military and maritime forces, and sailors belonging to the commercial marine, so that there was one death from consumption for 257, or 3·89 in 1000 inhabitants,—almost as great a proportion as in England.

An extended series of inquiries, put into a tabular shape, show that, of the deaths in the French army, amounting to 17,209, from 1820 to 1826, 1260 were from phthisis, giving a proportion of 1 to 13·6. Similar investigations, made with great care by M. Rochard, of men sailing from and arriving at Brest, show the mortality from phthisis, compared with the whole number of deaths, as 1 to 3·86,—a terrible commentary on the alleged sanative effects of sea air and sea-voyages in consumption. Similar estimates made of deaths in the Toulon public and commercial marine, exhibit analogous results to those just given of Brest, notwithstanding the situation of the former city in the Mediterranean, and its exposure to the southerly winds blowing from that sea,—circumstances supposed to be so beneficial to those with weak chests, and which would operate favorably on the sailors during their stay in port. Exemption from phthisis was claimed in favor of Cherbourg, which, like Brest, is on the Atlantic coast of France; but, as M. Rochard shows, without cause, either as regards the resident inhabitants or the maritime part of the population.

The author, anticipating an objection that might be raised against the above conclusions, on the ground that the great mortality among sailors from phthisis is due to exposures during their stay in port, shows that so far from this being the case, pulmonary tuberculization makes more rapid progress at sea than on land, and that death among the consumptive at sea is quite a common event. Summing up the number of deaths at the different French naval stations, viz., the West Indies; South Seas, Oceanica and New Zealand; India and China; Brazil and La Plata; and the Western Coast of Africa, which amounted to 691, in a force collectively of 82 vessels and 16,612 men, M. Rochard found that the deaths from consumption were 91, or 1 in 7·59 of other diseases, being nearly double that which occurred in the army. Taking the effective of all on board ship, it would seem that one consumptive sailor dies out of every 364, or 2·7 in 1000 on duty in a year; while in the army the proportion is as 1 to 578, or 1·7 in 1000. The reader should be informed that the French sailors of the national marine are, in some measure, a chosen class, as the men who labor under any special disease or infirmity are not enrolled. To a certain extent this remark is applicable to the English maritime force.

The author gives a statistical summary of the results which occurred in the different commands of the English navy (without specification of period) as he found them in the *Gazette Medicale*, 1841. We prefer turning to the "Blue Books" * themselves, in which we find the following figures, culled from the tables in the reports made up by Dr. Wilson. We must premise that the mortality in England from all diseases of the respiratory organs, in 1838, was 7·60 per thousand of the entire population, and from phthisis, 3·93 per thousand. In London, the deaths from the class of diseases first mentioned was 7·83 in 1000, and in the second, or from phthisis, was 4·14 in 1000. The deaths from phthisis in London were 234 in a 1000, or a little more than 23 per cent., or 1 in 4·2 deaths from other diseases. In the South American command or squadron, the mean effective strength of which, for seven years, was 2600 annually, the cases of phthisis or "consumptive diseases of the lungs" were 55, or 3·2 per 1000 of effective mean strength, and the deaths from phthisis 25 in the septennial period, or 1·5 per 1000 annually. The whole number of deaths from other diseases was 134, which would give to phthisis 18·6 per cent., or 1 to 5·36 from other diseases. In the West Indian and North American squadron, with an average effective force of 4907, the cases of phthisis were 114 or 4·8, and the deaths 44 or 1·9 annual ratio per 1000 of

* Statistical Reports of the Health of the Navy for the years 1830, 1831, 1832, 1833, 1834, 1835, and 1836, in two Parts. Part I. South American, West Indian and North American, Mediterranean and Peninsular Commands, 1840. Part II. Cape of Good Hope, and West Coast of Africa, and East Indian Command, Home and Various Forces, 1840 and 1841.

effective force. The deaths from this disease were to those from other diseases in the proportion of 10 per cent., or 1 to 9·7. In the Mediterranean and Peninsular command, the effective force of which was, on an average, for seven years 7958, the cases of phthisis numbered 285, and the deaths 106; the annual ratios per 1000 were 5·1 and 1·9, and the proportion of deaths in phthisis to those from other diseases amounting to 516, was 20·5 per cent., or 1 in 4·86, which approaches nearly to that of London. In the Home and Various Force, the vessels of which sail on the coasts of Great Britain and Ireland, the British Channel, North Sea, and St. George's Channel, and in the Atlantic Ocean to the limits of other commands, the cases of consumption, in an average effective force of 5391, were 156, or 1·6 per 1000 per annum, and the entire number of deaths from all diseases 359, of which phthisis numbered 60, being in the proportion of one of the latter to six of the former, and 1·58 in 1000 of effective force, which exhibits a more favorable proportion than in the Mediterranean.

In a table of totals throughout the service for seven years, in the three commands as above, not including the Home and Various Service, we find deaths from inflammation of the lungs set down as 1 per 1000 of effective force; from phthisis 1·6, to the same number, making 2·6 for diseases of the respiratory organs per 1000 of effective force, and giving a ratio which comes next to that of fevers. The deaths from these were 3·7 in a 1000. Consumption, counting 1·6, comes next to fevers in the proportion of deaths to the effective force. The ratio of cases is, in all the commands, 4·2. In that of the Mediterranean it was as high as 5·1. Contrary to *a priori* reasoning on the subject of marine climatic influences, we found that although the entire mortality from phthisis in the crews of the different vessels at sea falls short of that in all classes on land, yet that it is less in the northern and inclement latitudes of the North Sea and British and St. George's Channel and on coast service than it is in the Mediterranean, the climate of which was so long thought to be congenial to those with weak or diseased lungs. In the Home and Various Force, we see the mortality from consumption to be 1·6 per 1000, while in the squadron of the Mediterranean and cruising round the Peninsula (Spain and Portugal) it is 1·9 per 1000. "Inflammation of the lungs and of their membranes, and especially the interior lining the air-passages, is rather a prevalent form of diseased action in the Mediterranean, for which the nature of the climate, in regard to temperature particularly, sufficiently accounts. It is, as has been stated, very variable, the changes on the north coast being sudden, and often violent. When the wind shifts from north to south, and *vice versa*, a fall or rise of twenty degrees in the thermometer will sometimes take place in a few hours." Rep., Part I, p. 192. And again at p. 227, in speaking of the three "commands:" "But while these affections of the alimentary organs were less frequent and fatal than in the other two commands, organic diseases of the pulmonic apparatus were much more frequent than in either, and, including primary inflammation, more fatal, especially when compared with South America." Contrary to the vulgar notion, that sailors do not take cold, we learn that, even in what used to be regarded as the favored climate of the Mediterranean, seafaring men are peculiarly liable to catarrhal affections, and to "primary inflammation of the pulmonic tissues, embracing pneumonitis, pleuritis, and bronchitis." Rep., Part II, p. 215.

The author of the reports, Dr. Wilson, thinks that they "establish the important fact, that sea life has a sanatory influence on non-inflammatory affections of the lungs, at least on the most destructive one of them, the phthisical. The influence is chiefly preventive, but would appear to be also in no small degree curative." A qualifying remark, however, soon follows, not calculated to make us place much confidence in sea life as a remedy for consumption. "The amount of curative agency cannot be determined; but it is feared that it cannot be considered much." This is the conclusion to which we are afraid we must arrive, when we reflect on the fact, that the liability to attacks of inflammations of the lungs and their membranes in seafaring men, subjects those predisposed to phthisis to a development and aggravation of this disease when it is present. We all know the bad effects of either preceding or intercurrent inflammation of the lung in the phthisical. In the year 1833, consumption was the most fatal disease in the Mediterranean squadron, not even excepting malignant cholera, which prevailed in some of the vessels. The same remark may be made of the same force in 1832; and if we include all the diseases of the lungs under one head, they will be found to make up more than one-half of the entire mortality.

The above results correspond with those announced by Dr. Sinclair twenty-seven years before, in his Inaugural Thesis, published at Edinburgh, 1817. He gives the hospital returns, in 1810, 1811, and 1812, of the diseases of the seamen on board the British fleet, numbering about thirty thousand men. During the above period, there were admitted into the hospitals at Malta, Gibraltar, and Minorca, 2113 patients, of whom 596 were laboring under phthisis pulmonalis and pneumonia, so that the rate of pulmonic to other diseases was as 1 to 2·5. The rate of mortality in the two was, however, very different. Out of 455 cases of phthisis alone, 151 died before the remainder could be shipped off for England, where, in all probability, most of them perished. Whereas, out of 1242 cases of fever, only 58 died, and a very small number were invalided. The preceding figures would indicate a proportion of cases of phthisis to the effective force of 30,000 men, as 5 to a thousand in a year, and the deaths in the hospitals, without adverting to the fate of those sent home, as 1·6 to 1000 of effective force per annum. This mortality hardly bears out Dr. Wilson's belief, which he indicates in the form of a question, that the deaths from this disease are not so numerous as they used to be in the crews of public ships. But if we take the entire number of deaths in the hospitals and of those sent home, it cannot have been far short of the number of cases, or 5 per 1000 effective force. The reader must also be apprised of the deteriorating influences at work on sailors in the years above mentioned, in the way of continued confinement to their vessels, and subjection to all the injurious effects of bad quarters in old vessels, and of a want of change of food,—it being a time of war. Causes of this nature are still, but in less degree, operative on seamen at the present time, who are also, of necessity, exposed to the chilling and damp air of the night in their watches on deck, as well as to great transitions, by leaving their close-confined quarters between decks, and coming up into the open air. But, on the other hand, the large compensation which they enjoy from their spending so much of their time in this free and pure air, and the, in general, regularity of life at sea, added to the greater average strength and vigor incident to their ages, and kept up, for the most part, by abundant alimentation, give them decided advantages over the population at home and on land, which furnishes the largest number of cases of phthisis.

So far as we can deduce conclusions from official reports of the vital statistics of the British navy, they would seem to indicate a difference in favor of maritime life over that on shore, as regards the number of cases of phthisis and the deaths from this disease. The proportion of deaths from phthisis to those from all other diseases is, however, greater at sea than on land, if we except the South American command. It is this fact which seems to have led M. Rochard to the inference, that there were actually a greater proportionate number of cases of phthisis and resulting mortality on board ship than on land.

A comparison between the military and maritime forces is in favor of the latter. Thus we learn, from official returns contained in the Parliamentary Blue Books, that the cases of consumption among the British troops in garrison, on an average of twenty years, were 6 per annum in every 1000 men. In the Ionian Islands, the proportion is 5 in 1000, which is slightly less than that among sailors in the Mediterranean. In an aggregate strength of 86,661 British soldiers, during a period of nineteen years on the Windward and Leeward Islands, the deaths were 6803, of which 906, or 1 in 7·5 were from pulmonary diseases, and 580, or 1 in 12, from pulmonary consumption. The deaths from this disease, in the military hospital at Jamaica, was 6·1 in 1000 of the mean strength. In London the deaths were 4·14 in 1000 of the inhabitants.

A fact stated by Dr. Wilson, in the Report, Part I, p. 192, is adverse both to the sanative effects of sea-voyages and of warm climates: "It is ascertained, though the fact is opposed to many preconceived opinions, that bronchitis, which originates in the Mediterranean, and which often runs rapidly to a fatal termination there, may be arrested by moving the subject to England. A similar remark was made respecting the disease in South America." Dr. Sinclair had previously placed in contrast the effects of a winter in the Mediterranean and one passed far north, to the credit of the latter. In the severe winter of 1814, the Colossus ship-of-the-line, of which he was then surgeon, remained the greater part of the season in the river Scheldt, inclosed in the ice. During this time, Fahrenheit's thermometer continued much below the freezing-point for several weeks, as low even as 18°, and yet on this occasion the number of men attacked with catarrh and pneumonia was seldom so great as he had witnessed during the preceding summer and autumn, in the Mediterranean, among a crew of only half the number (three hundred).

Officers of the marines, who, on board their ships, are placed in the same situation as passengers would be, and who are. not exposed to rain and storms as the sailors are, pay, M. Rochford assures us, the same tax with these latter, in a certain number of them being carried off by consumption. From all that precedes, one conclusion of considerable moment may be drawn, viz., that young persons with delicate chests and predisposed to pulmonary tuberculization, ought to be advised against going to sea. Hitherto, the advice has been given the other way.

Is sea air favorable to the consumptive? As relates to living entirely at sea and breathing a sea air, the question has been already answered. We have now to speak of residence on land with a maritime exposure, and breathing to a certain extent a sea air. By many this is thought to be beneficial. What will they say when told that a sea air is breathed by the people who inhabit the maritime districts of Great Britain and Ireland, and of Holland and Germany, and the Atlantic seaboard of the United States, in all of which regions consumption prevails to a great extent? The general proposition is then qualified with a condition that the maritime exposure be to the south or the south and west; but even this qualification will not meet the case of the maritime belts of Holland and North Germany. As we have in part learned already, and shall show more fully hereafter, the desired exposure, in all its amplitude, will not secure a sanative resting-place for the consumptive.

Some stress has been laid on sea sickness as a cause explanatory of the good effects of sea-voyages in phthisis; but the cause is too ephemeral, and what is more to the point, its good effects are yet to be proved. A change in the manner of life is also regarded as exerting a hygienic agency of some power, in substituting sea for land travel. There can be no question but that the change of scene, great at first, and which, afterwards, must count for something, regularity of meals and in the hours of sleep and waking, all passionate excitements withheld, and yet still room given for intellectual exercise without intense occupation—benefits similar to those procured by visits to mineral springs, sea bathing, and country life—must be productive of beneficial results. During a long voyage, such as to the East Indies or to China, or round Cape Horn into the Pacific, time would be allowed for salutary effects of this nature, which are quite out of the question in our common transatlantic passages, especially in steamers.

*Emigration to Warm Climates* heads a chapter in M. Rochard's memoir. Incidentally, we have already touched on some parts of this inquiry, when speaking of sea life in the Mediterranean and in the West Indies, and of the effects of the climate of these regions on soldiers, as relates to a tendency to consumption. The author, in reference to the effects of migrating from a warm to a cool or cold climate, reminds us, that the negroes of Sennaar become tuberculous in Egypt, as noticed by Clot Bey. In the islands of Ceylon, Bourbon, and the Mauritius, phthisis commits great ravages among the negroes from the western coast of Africa, as it also did among the soldiers of the black regiment in Gibraltar. Further north the race suffers still more from this disease. But the change which more directly concerns us is the migration or movement from cold or temperate, to warm or hot climates. We have seen the great frequency of catarrh and bronchitis in the Mediterranean, to which region it is still the fashion to send persons suffering from these diseases in their chronic state, and from phthisis itself. Changes of temperature and wind in southern latitudes exert a very prompt and sinister operation, especially in those from the North, who are affected at once with catarrh and bronchitis, or with intestinal irritation and flux, according to the season and their predisposition.

Tuberculization makes the greatest progress in hot climates, without the impulsion, as it were, given to it in our northern climates by catarrhal irritation or by bronchitis, pleurisy, and pneumonia, each of which may, and often does, serve in its turn to accelerate the progress of the consumptive to the tomb. M. Rochard cites the opinion, based on experimental observations of French physicians and surgeons, in proof of the accelerated course of pulmonary tuberculization in the French West India islands of Guadaloupe and Martinique, in Cayenne and Senegal, and in Hindostan and the islands of the Southern Pacific. Not only do the physicians in the French colonies protest against the phthisical being sent there, but they recommend strenuously the departure of those among them who are affected with phthisis, to return to Europe. As relates to the army, both French and English statistics agree in showing the fearful ravages committed by phthisis pulmonalis among the troops of both countries in tropical and warm latitudes, exceeding in this respect the mortality from this disease at home. In the Tulloch's English Army Reports, speaking of the West Indies, we read the following: "As an instance how much more prevalent consumption is in that country than in Britain, we may state, that of an aggregate strength of 57,567 serving in Jamaica, there have been 661 treated for that disease, being at the rate of 13 per thousand annually; while out of an aggregate strength of 44,611 dragoon-guards and dragoons, serving in the United Kingdom, there have been treated only 286, or between 5 and 6 per thousand annually,—and that, too, though the period over which the latter observations extend includes two severe epidemics of influenza, which no doubt laid the foundation of more cases of this disease than usually occur in this country." (See also *anté*, p. 817.)

These morbid peculiarities are not confined to the military. Chisholm had, many years ago, pointed out the fact of the frequent occurrence and rapid march of the disease (galloping consumption) in Granada. Cornuel gave similar testimony for Guadaloupe, and Rufz for Martinique. There are deterioration and anemia of the organism in the phthisical in hot climates. They do not digest well, nor make good blood to resist tuberculization, and bear them up against its depressing and enfeebling operation. Brazil, so lauded for its balmy air, pays its full tribute to consumption, both at Rio Janeiro and in the provinces, as we learn from M. Sigaud; and another writer in the *Gazette Médicale* states that the number of consumptive patients in the hospital of that city is nearly as great as in Paris. One of the professors of the University in Rio expressed his belief that a sixth part of the mortality among the poorer classes in the Brazils was due to phthisis.

M. Rochard next passes in review the division of the warm climates to the north of the tropical zone. It includes a region between the tropic of Cancer on the south, and the isothermal line of 15° C., equal to 59° F., on the chart of M. Boudien, on the north, and which corresponds with the forty-fifth degree of north latitude in the old world, and the forty-first in America. It includes Spain, the south of France, Italy, Greece, and the basin of the Mediterranean, Madeira, the Canaries, the kingdom of Morocco, Algeria, the regencies of Tunis and Tripoli, Egypt, the north of Arabia, Syria, and Turkey in Asia. In America it includes the Southern States of our Union, Mexico, and California. An immense space this from which to select abodes for the sick, and especially those with weak lungs; and we might suppose that there could be no difficulty in finding many spots favored by a bountiful nature, if not always embellished by art. Were we to judge from the literature of the subject, there would seem to be numerous guides to point the way, and to tell the very degree of temperature and hygrometricity, the precise extent of perflation, and the kind and amount of electricity required by the invalid, to be found at the spot chosen in the exercise of their ingenuity, or the display of their learning. There are, however, writers on the subject who have brought to its investigation personal experience and considerable observation, and whose inquiries have been aided by liberal-minded physicians of the place described. The summing up, unfortunately, after putting aside all that is foreign and collateral, and not sustained by very close analogy, includes little that is clearly positive and mandatory, or deducible from direct indisputable evidence. But the ground has been gone over at different times, and it will be again; and it is not for us, who profess to sweep the horizon with a critical eye, to turn away in discouragement or despair.

The works, the titles of which head this article, now come before us with a common purpose, and their authors use, very often, the same materials; for even Dr. Taylor, while professedly treating of the climate of Pau, engages in a critical comparison of the hygienic value of this place, with that of other noted health-praised resorts. M. Carrière, under the title of the Climate of Italy, introduces many observations on comparative climatology. In fact, while we find in his work continual evidences of an observing and a thoughtful mind, we could spare some of the disquisitional portion of its pages, without any diminution of its utility. Mr. Lee's Essay is a well-written summary of the etiology of pulmonary tuberculosis, and of the influence of climate in retarding and ameliorating this disease, followed by an appendix, which contains a *Notice of some of the Places most frequented on account of their Climates in the South of France, Italy, &c.* To say that this essay contains nothing new, or that it might be illustrated by some additional facts in pathology and animal chemistry, would imply a minute criticism, applicable to almost any of the prize essays of the day. A more just, and to us more pleasant way of looking at it, is as a work to which reference may be instructively made by readers who have not studied the subject, or who have given it but a cursory notice. The author begins with "*Preliminary Remarks*" on the curability of consumption, and gives proofs derived from numerous *post mortem* examinations of aged persons, to which he might have added the entire restoration to health of invalids, who, as ascertained by careful physical and physiological diagnosis, had undoubtedly labored under confirmed phthisis. Faith in such a result leads physicians in Great Britain and the United States to reach it by pharmaceutical means, to the neglect, if not exclusion, of those hygienic agencies, steadily and perseveringly applied, which might alter the intimate condition of the organism, and change, as the phrase is, the constitution, so that a permanent cure might be obtained instead of temporary palliation. It is well remarked by M. Baumes, as quoted by Mr. Lee, that "to occupy one's self in treating merely the manifestations of a diathesis, is generally as if one were to run after the shadow, and leave the substance, which it is desirable to obtain." A difficulty, however, presents, in not knowing very accurately when the curative treatment should be begun, owing to a want of correct diagnosis in the early period of tuberculosis; for, although there are very precise directions and rules to be pursued in these cases, they are not of easy adoption without some natural tact, and continued trials and experience; and even if we suppose all the desired attainments on the part of the physician, the attention of the invalid is often not awakened to his own condition by any positive pain or distressing symptom, until the disease has made some progress. A stage is at length reached when no doubt remains, and when art has tried all its resources in vain. The patient tired and the physician tired, travel is suggested; and the prospect is held out by sanguine friends, perhaps by the medical attendant, of marked amendment, if not restoration to health. Buoyed up by hope, the invalid revives as his itineracy is laid down; and he sets out almost rejoicing, and counts the time when he is to return home in the plenitude of health. He reaches the abode of Hygeia, and sometimes revives for a season; but he is too easily content with this first instalment, as it were, of a larger stock of health in reserve when he reaches his native land. He then hastens back, sometimes with such impatience and prematureness, as to cause him to lose, by the keen winds and vicissitudes of spring, all that he had gained during his winter residence abroad. Others again, and they are unhappily too numerous, leave home when it is too late: the disease is in its second, if not third stage, and they go to a foreign land—to die. It is necessary for us to bear these things in mind, if we would have our patients derive any substantial benefit from travel and change of climate; and also if we would appreciate at their true worth the sometimes contradictory and conflicting statements of the sanative effects of certain spots, which are differently regarded according to the stage of the disease from which the experience is derived.

Dr. Lee's second section is "*On the Nature of Pulmonary Tuberculization.*" He speaks of, without adopting unreservedly, the opinion of several distinguished pathologists, who have considered tubercle to be a product of inflammatory action. Among these he mentions Broussais, Bouillaud of Paris, Addison of London, Reinhardt of Berlin, and Van der Kolke of Utrecht. On the adverse side we find the names of Laennec, Bayle, and Louis. The more correct is the modified view taken by Carswell, Andral, Fourcault, and Clark, who, while they deny that tubercle is a product of inflammation, believe that this process, or even an irritation, may by attracting the morbid deposit and developing tuberculosis, so far prove a determining cause. The prevailing, and, we must needs think, the correct opinion at the present time, is to regard tuberculization as a disease depending on an alteration of the blood from its normal condition. It is easier, however, to believe in this morbid change than to show distinctly in what it consists. The experiments of MM. Andral and Gavarret are valuable contributions to this end. A point of great importance, inasmuch as it suggests timely preventive means, is the undeniable fact, that the morbid state of the blood which causes tuberculous cachexy, may exist for a long time before the formation of tubercle itself in the lungs. The more immediate cause of the vitiated state of the blood is to be found, the author thinks, in the inactivity of the capillary circulation of the skin, and the consequent impediment to the regular and free discharge of insensible perspiration, and to the elimination from this surface of effete matters from the general system, and particularly from the blood. Nearly, if not quite of equal importance, is the regularity of its function, as auxiliary to the lungs in respiration, by the evolution of carbonic acid, and, to a certain extent, the absorption of oxygen from the atmosphere. In this the skin, according to its healthy or morbid state, will materially aid or materially injure the lungs, not only in their healthy, but also their morbid condition. Experiments by Ingenhous, Cruikshank, and Abernethy, are referred to in connection with this point. Still more to the purpose are those of Fourcault.

The next step taken by Mr. Lee, is an inquiry into the *Causes of Pulmonary Consumption*, if, indeed, we can refuse to admit what has just been said to be a part of the etiology of the disease. Certain it is, that these pathological premises prepare us for an appreciation of certain atmospherical conditions, such as humidity and brisk transitions of temperature, among the external causes of phthisis, and the absence or the free indulgence of exercise in the open air, as respectively a cause or a preventive. We repeat, without giving it full credence, the following opinion of the author. "The comparative exemption from consumption which the inhabitants of cold and dry countries enjoy, is attributable, in a great measure, to their greater activity, in order to guard themselves against the effects of cold." Dr. Foissac, in his recent work on Meteorology, quotes the remark of Dr. Wrangele, that "Diseases are of rare occurrence in Siberia, and old people preserve their vigor until a very advanced period. The exercise which they take in the open air, whether travelling on sledges or skating over the ice, is the chief cause of their good health." Pulmonary consumption, and tuberculous diseases in general, are especially frequent among young persons, or females who lead an inactive life, and hence it bears with peculiar severity on seamstresses, turners, &c. An active life, or active mechanical employments will, to a great degree, mitigate, if not nullify the depressing and deteriorating effects of humidity. With sedentary life and sedentary employments are generally associated defective ventilation, by which both lungs and skin suffer, unable as they are to quicken their functional exercise, and throw off the deleterious elements.

These being retained, poison the blood, and through it depress and deteriorate the nervous system, and thus almost insure the formation of tubercle, especially where, by inheritance or early weakness of frame, a predisposition to the disease exists. Ancell, Guy, Lombard, and others, have collected valuable statistical information on this head.

As connected with the effect of the impression of cold or dampness upon the surface in inducing tuberculous disease, Mr. Lee points out the neglect, the criminal neglect we would term it, of wearing clothes of such a texture and fashion as might protect the skin against the frequent vicissitudes and great extremes of temperature, so common in the climate of the United States, for on this point we may generalize, and of Great Britain. Females and children are the greatest sufferers in this way,—the former punishing themselves by yielding to the silly requirements of fashion; the latter punished by their mothers, and sometimes by their weak-minded fathers, who dress them out as if they were show-dolls in a shop window, to attract the passers-by. Exposure of the neck, chest, arms, legs, and feet, to cold and humidity, in these two classes of persons, tends to depress the powers of life, and to develop into a dangerous and fatal disease, a predisposition which, with a better system of physical education, might have been dormant for life. The absurdities of fashion bring the rich on the same level with the poor, in the privation of suitable covering and warmth for the body. We are surprised at Mr. Lee's omitting to mention and to press a practice of the highest importance, directly deducible from the facts previously stated, which show the necessity of preserving for the skin the full exercise of its functions, both as an organ of respiration and one of depuration. We refer to regular ablution of the entire surface, by the use of the bath, and to friction as accessary to bathing, besides that it imparts a healthy excitement to the skin. Excuses may be found for the poorer and less favored classes neglecting their balneatory duties, owing to the want of facilities for bathing, although there is no bar to a good daily washing of their skins: the process is of easy accomplishment, wherever there is a wash-tub, a bucket and water, and a corner of a room for one's privacy during half an hour. But what shall we say of the neglect of regular bathing on the part of the rich, and those in easy circumstances? Very many who would resent as an insult any imputation of their being uncleanly, and who shrink at the idea of wearing soiled linen, seem to have no dislike or dread of a perpetually soiled skin; coated for days, and weeks, and months, with the perspirable and other exuded matter, which has been allowed to dry and accumulate, without any attempt on the part of its owner to remove it by a bath, or its partial substitute, good daily friction with a flesh-brush or coarse towel.

Mr. Lee, while adverting to, errs in underrating the importance of wholesome food and good digestion, as one of the preventives of pulmonary tuberculosis. It is true that the dyspeptic in large numbers escape consumption, unless they have been predisposed to it; but it is not the less true that scanty diet must contribute largely to cause weakened digestion, and fail to furnish sufficient pabulum for complete hematosis.

"The effect of depressing passions and emotions has been" (Mr. Lee thinks) "too little considered by medical practitioners as strongly tending to tubercular cachexia." Of the interference by such perturbations with nutrition there can be no doubt, nor, as a consequence of their prejudicial operation in consumption. But yet, we remember in our youthful studies, to have read that a sedation of this kind was actually recommended in the disease, with a view to diminish the morbid activity of the circulation, which was supposed to keep up the symptoms, as measured by the frequency of the pulse. That was the age in which Beddoes told the world that digitalis would one day be regarded as exerting a controlling power over hectic fever, similar to that possessed by Peruvian bark over intermittent fever. Days of hope those—days of warning to us, that we may not pin our entire faith on cod-liver oil and iodide of iron now, as Beddoes and others did then on digitalis, the inhalation of factitious gases, and living over cow-houses, so that the invalid might breathe the air made balmy with the exhalations from the quadruped tenants.

The question, and it is a very important one too, of the marriage of the consumptive, or of those predisposed to consumption, is passed over by the author as foreign to the subject which he has undertaken to treat. It is true, as he justly remarks, that the principles of the prophylactic and curative treatment by climate and other means, "would be about the same in both cases;" the inherited predisposition and the adventitious disease.

The main topic of Mr. Lee's essay is reached in the fourth division, *The Effect of Climate on Tubercular Consumption.* Individual differences of constitution and temperament, and the stage of the disease, will modify not a little the views which we may entertain respecting the curative effects of the climate of particular localities. Among the more prominent conditions for the invalid to derive the greatest benefit by residence, for a season, in any one of them, are protection from great extremes and sudden vicissitudes of weather, and opportunity for being out and exercising much in the open air, without his running much risk of contracting catarrh, or, worse still, a pleuritic affection. It is almost equally necessary, on the other hand, that the climate should not exhibit a constant sameness, which, apart from its unfavorable physical effects, would induce a wearisome monotony of the mind and feelings. A uniformly calm atmosphere, scarcely disturbed by winds, or agitated by even a breeze, comes next to a stagnant atmosphere, and is detrimental to the full exercise of the respiratory apparatus, as well as to that of the cutaneous functions.

Let us now inquire briefly into the respective advantages of the most known and celebrated retreats and resorts for invalids, who leave home with the expectation of being benefited by a change of climate. We shall not presume on any experience personal to ourselves on this occasion, for although we have visited some of these places famed in story, before they were thought of hygienically, yet our stay was too brief to allow of our gleaning any information of moment on the main subject. Of the guides on our table, M. Rochard displays the most scepticism, Mr. Lee judicious selection of opinions, Sir James Clark caution in his praise and explicitness in his doubts and dissents, and M. Carrière fulness of detail and explanation, with an occasional blending of fact and speculation. We pass over Spain and the Balearic Islands, to light on France; and even in *la belle France*, we shall turn our backs on Montpellier and Marseilles, and casting a side glance at Aix, as one might be expected to do at a discarded favorite, we stop for a brief period at Hyères, which, according to M. Rochard, is the only spot in the south of France adapted to consumptive invalids. Hyères is only four leagues distant from Toulon; but it is separated from that city by a chain of hills, on the southern slope of which it rises, and is thus in a degree protected from the mistral, although at times this wind, the northwest, blows over it with considerable force. "Of sufficient elevation, and at a due distance from the sea to place it out of danger from the salines on the coast, and lost in the midst of orange groves and hedges of laurel roses, Hyères constitutes the most charming abode that could be selected by an invalid." M. Barth has given an inviting picture of this place, and, adopting his opinions, we should proclaim Hyères, with its mild and pure air, as admirably suited to cases of pulmonary catarrh, chronic pleurisy, and pulmonary emphysema. But when it comes to the effects of this air on phthisis, M. Rochard holds back from his countryman just named. "The cemetery of Hyères is stocked with the bodies of the consumptive, and death spares but a small number of those who have gone to it for a retreat from danger." A great many phthisical subjects, says M. Andral, visit this place every year, not to obtain a complete cure of their disease, but a prolongation in a greater or less degree of their existence. Is not this, in a few words, an opinion applicable to all the places of resort for invalids on the shores of the Mediterranean? But duty forbids us to shut out hope, and we continue our health quest. Sir James Clark, writing of the place, tells us: "When the weather does permit, the invalid residing at Hyères may enjoy the advantage of a variety of rides through a fine open country. But when the mistral wind blows with any degree of force, he should confine himself to the house if his chest be delicate; and he must be cautious at all times of exposing himself to this strong wind, which, independently of its low temperature, is very irritating. With all these objections, the climate of Hyères is the mildest in Provence."

We cross the French frontier and are in Italy, in the dominions of the King of Sardinia. The city which the first attracts notice on this route, and to which a large number of individuals annually resort, is Nice. Geographically considered, the territory on which this city stands is a continuation of Provence, the climatic character of which it closely resembles. The temperature throughout the year is more equally distributed at Nice than at any place in the south of Europe of which we have accounts, except Rome and Cadiz; the difference of the warmest and the coldest months being only 28°, and the mean difference of successive months only 4·74°. The range of temperature for the day is also less at Nice than at any other part of the south of Europe; and in steadiness of temperature it ranks next to Madeira.

The mild and agreeable temperature of Nice, continues Sir James Clark, depends, in a great measure, on the position of the place with respect to the neighboring mountains and the sea. The maritime Alps form a lofty barrier, which shelters it from the northerly winds during winter; and the cool sea breeze which prevails every day, with a regularity almost equal to that of a tropical climate, moderates the summer heat. The same writer, in stating its influence on the animal economy, would say, that the climate of Nice is warm, exhilarating, and exciting, but upon the whole irritating,—more especially during the spring,—at least to highly sensitive constitutions. It is extremely favorable to the productions of the vegetable kingdom, some of which flourish here in a degree of luxuriance that is scarcely to be equalled in the other parts of the south of Europe.

The dryness of the air of Nice, so generally asserted, is denied by M. Carrière, who gives a detailed description of the topography and climatic characters of this place, which, if a pleasant winter, is far from being an agreeable or a safe spring residence, owing to the predominance of sharp easterly winds in the latter season. Hence, invalids are advised to make their escape to a more southerly spot in Italy before the spring sets in. Lymphatic constitutions, and relaxation and torpor of the system generally, are benefited by the climate of Nice. This will include atonic dyspepsia, irregularities of the menstrual functions, with asthenia and anemia. In scrofulous complaints, in a remarkable manner, and in gout and chronic rheumatism, residence in Nice confers benefit. Humid bronchitis is greatly relieved by a winter's stay here. The same cannot be said of phthisis, which, Sir James Clark tells us, will, he fears, be little benefited. M. Rochard goes farther, and dismissing Nice in less than a page, quotes Dr. Pugh's melancholy account of seven persons, six young men and an old lady, consumptive subjects, finding their graves there in the course of a winter. Of the six males, Dr. Pugh firmly believes that four would have had their lives prolonged by a return to England, or by withdrawing to the south of France. Bricheteau says that in the hospital at Nice, one in seven of the deaths is from consumption.

Nice, with a population of 40,000 souls, has variety and amusement sufficient to prevent time from hanging heavy on the invalid. Two libraries with reading-rooms, a *circle* with a good library, and French, English, and Italian papers, a tolerable theatre, &c., offer resources in the city, while excursions in the country around, open to the eye a pleasing diversity of scenery and concomitant pleasures. The invalid who wishes to escape early in the spring has the road to Genoa open to him.

Menton and Villa Franca, the last of which is separated from Nice by a high hill, deserve, in the opinion of M. Rochard, to be much more highly thought of in consumption than Nice itself.

Genoa is not a fit residence for the consumptive. The same must be said of Florence,—attractive, enchanting Florence,—where learning and the fine arts so long have had their abode, and where still the man of study and of taste must love to make a long stay, even though it were to be for life. But the *poitrinaire* most carefully shun our favorite Florence as a winter residence, exposed as it is to the keen blast from the Apennines, at the base, or rather encircled by the spurs, of which it lies. This city is one of the places in Italy which agrees the least with children.

Pisa, the almost neighbor, and in her republican times, once the rival of Florence, has stronger claims on our notice as a winter residence for invalids. It is on the banks of the Arno, six miles from the sea-shore, fifteen from Leghorn, and about forty-five from Florence, a distance now travelled by railroad. Two ranges of hills shelter it in a measure from the northerly and the easterly winds. The best residence for invalids is the *Lung d'Arno*, a range of buildings in a crescent form, corresponding with a semicircular sweep which the Arno makes to the north, so that a broad space left between them and the river is in greater part sheltered from the northerly winds. This is the preferred residence for invalids, who can sun themselves while taking a stroll along the quays: these, together with the houses, and the cathedral, baptistery, and campanella, tell of the wealth and splendor of other times. The climate is genial, but rather oppressive and damp; the latter property is kept up by the great quantity of rain which falls,—45·66 inches,—and by the marshes, which extend to the west and south as far as the sea-shore.

Pisa has an advantage over both Nice and Rome in its being better sheltered than they are from cold winds. Invalids, who dread the easterly winds of spring at Nice, sometimes take shelter for a while during this season at Pisa. By the soothing and almost sedative character of its air, it contrasts with Nice and Naples, and is a good residence for irritable systems with a tendency to phlogosis, which would suffer from a stay in the cities just named.

Venice, of which M. Carrière gives an interesting topographical description, resembles Pisa in its moist and mild climate, and hence it is, like the latter, adapted to phthisis and other chronic affections of the respiratory apparatus. It has an advantage over most other places in Italy as well as those in the south of France, in the longer period of the year in which invalids can remain without suffering from heat. They may take up their quarters in Venice in the autumn, and prolong it to the end of spring. The best quarter of the city to spend the winter in, to enjoy a special climate, as it were in another climate, is, at the same time, the finest, the most brilliant, and the most animated, viz., that of the Square of St. Mark and the Piazzetta, and along the chief part of the great canal to the wonderful bridge of the Rialto and the quay of the Slavii, which extends from the palace to the eastern point of a triangle. There the traveller finds an oasis of verdure, planted, as a Frenchman is proud to tell, by French hands.

Rome, situated on a basin intersected by the Tiber, is open to the dry, cold, northeast winds from the Apennines, and to the warm, moist, southerly winds from the Mediterranean and Campania. Its climate, on the whole, is mild and soft, and the air still; high winds being comparatively of rare occurrence. As Sir James Clark well observes: "This quality of calmness is valuable in a winter climate for pulmonary diseases, and to invalids generally, as it admits of their taking exercise in the open air at a much lower temperature than they could otherwise do. To patients laboring under bronchial irritation, wind is particularly hurtful." It is only the plethoric and healthy who suffer from the *sirocco*, or warm wind of the south. The critical season for invalids is the spring, or in the months of March and April, when the cold winds set in,—generally in the forenoon, and continue till sunset, when they subside, leaving the nights calm and serene.

Rome, a refuge to the consumptive invalid and to one who suffers from bronchial affections and chronic rheumatism, is itself, in the latter part of summer and in the autumn, liable to periodical fevers, often of great violence: they are called by the physicians there *pernicious;* by us, as we see them in our own country, *congestive.* Another and almost peculiar feature in the Roman air is the excessive sensibility of the inhabitants, as evinced in a disposition to convulsive affections, and a singular susceptibility to the impression of odors by the females of Rome. Another class of diseases of a much more serious character, and apparently, like the last, of modern origin, is apoplexy, and other cerebral affections, and also diseases of the heart and great vessels. Inflammatory affections of the lungs rank next, and appear to be more violent and rapid than in northern climates. Tubercular consumption is not common, and occurs chiefly among the poorer classes, who are badly lodged, fed, and clothed.

Sir James Clark specifies consumption as a disease which is benefited by a residence in Rome, not only by the allaying of present disease, but by preventing the development of tubercle, especially in young and strumous habits, the class most in danger from such a change. Allusion has been already made to the good effects of the Roman climate in bronchial affections and in chronic rheumatism.

The invalid of the commonest sensibility to the wonders of art, either ancient or modern, and who is ever so little instructed in ancient history, must find continual pleasure in visiting the spots illustrated by valor, patriotism, and genius, and the collections of art in painting and sculpture. He must be careful, however, how he exposes himself in many of these visits to cold churches, ancient baths, &c. St. Peter's Church is perhaps the only spot in which, on account of the nearly uniform temperature of its vast aisles throughout the season, he can linger with safety. The rides around Rome are numerous and are moreover diversified by the scenery, and the classic recollections to which they give rise.

The usually pleasant picture of Rome as a winter residence is greatly shaded by the most recent sketches from the pens of the medical staff attached to the French army of occupation in this city. Thus we learn, from the observations of M. Journé, quoted by M. Rochard, that, in the Hospital of St. John of Lateran, of 2540 females admitted, from 1834 to 1836, 126 were attacked with phthisis, and that in the same space of time, out of 379 deaths, 110 were due to this cause; giving the frightful proportion of 1 of the latter to 3·25 of the former, a ratio only exceeded by Naples. The French troops suffered, also, more from phthisis than the same number of them do in France; it being in Rome 1 in 9·71, while in the latter country we have seen that it was 1 in 13·6. Typhoid fever and consumption are represented to work together with fearful effect in Rome.

Naples, so full of attractions to the traveller in health, is destructive to weak lungs, as is evident by the frequency with which phthisis claims its victims. The transitions of weather are frequent and considerable, and the daily range of temperature great. In the words of Sir James Clark, "Naples is altogether an unsuitable residence for pulmonary invalids." According to the tables of M. Journé, the deaths from phthisis, in the civil hospitals, are to those from all other diseases as 1 to 2·33, and in the military, as 1 to 3·85: both higher than in London. M. Carrière enters into many details of the topography, climate, and soil of Naples, its past and present reputation as a residence for invalids, and certain prospective advantages which improvement in the sanitary condition of the districts of Pozzuoli and Baiæ may offer. He lays stress on the exciting properties of the air of Naples, by which it is particularly deleterious to the nervous and the irritable, to the extent of causing insanity itself. On the other hand, hypochondriasis may here be made to forget its double wearisomeness; that from which its possessor suffers, and that which he inflicts on others: there, also, melancholy may be made to smile and expand its gloomy brow.

Malta, sometimes mentioned among the climates of the Mediterranean, deserves no special notice in connection with our present theme. Consumption is frequent there, and runs its course with greater rapidity than in England.

Of Sicily our knowledge is too imperfect to allow of any satisfactory attempt at describing its local climates.

Algeria is represented, by the French army physicians of that country, to be the promised land of the consumptive. It would seem that there is less consumption among the soldiers there than in France. We must wait, however, for further revelations before we venture on any positive conclusion. How many paradises have there not been announced for the residence of the consumptive, and yet how few, if any, retain their original character.

Madeira, classed by Sir James Clark among the Atlantic climates, offers many inducements to the invalid to visit it, and to spend not only the winter but the entire year on the island. It is so easy to change the climate by ascending from the maritime belt, and thus, after spending the winter in the last-mentioned district, to procure a summer abode of any desired temperature according to the elevation that is chosen. Still, Madeira forms no exception to all the climates hitherto spoken of, in its failing to avert the progress of confirmed tuberculosis. In the first stage of disease it would seem, however, to exert a really restraining power.

The extension given to the present article entirely precludes us from noticing even, and still more from investigating, the different alleged health-giving spots in the United States and the West India Islands. The subject is of an importance and extent to demand a separate review of all its bearings and promises. We should be glad to see this taken in connection with a special notice of the chapter on the "General Sanitary Relations of the United States Climate" in Mr. Blodgett's volume.

There is yet another point on which some emphasis is laid by more than one of the authors, whose works we have endeavored to analyze on the present occasion. It is a recourse to mineral waters, as ancillary to the influence of climate in pulmonary affections. This question more properly belongs to the hygienic course to be prescribed for the guidance of the invalid during the summer, so as to insure a certain degree of harmony of treatment throughout the year, and a continuance of the improvement gained in one season through the next. But this field of observation must be trodden at a future time.

